# Psychiatric admission among migrants: a retrospective study in acute psychiatric ward in Bologna, Italy

**DOI:** 10.1192/j.eurpsy.2022.1400

**Published:** 2022-09-01

**Authors:** M. Galatolo, R. Biagini, G. D’Andrea, M. Farruggio, A.L. Carloni, G. Iuzzolino, D. Allegri, C. Descovich, R. Muratori, I. Tarricone

**Affiliations:** 1 University of Bologna, Department Of Biomedical And Neuromotor Sciences, Bologna, Italy; 2 UNIBO- ALMA MATER STUDIORUM - UNIVERSITA DI BOLOGNA, IT, Dipartimento Di Scienze Mediche E Chirurgiche, Bologna, Italy; 3 AUSL Bologna, Department Of Programming And Control, Bologna, Italy; 4 AUSL Bologna, Department Of Mental Health, Bologna, Italy

**Keywords:** migration, psychiatry admissions, cultural psychiatry

## Abstract

**Introduction:**

Numerous evidences point out how migrants use health services differently than the natives. Migrants turn more frequently to the ED for psychiatric problems and less to territorial psychiatric services than the native population. Other differences can be found in terms of diagnosis, type of discharge, type of hospitalization.

**Objectives:**

Our study has the objective of evaluating the incidence of psychiatric hospitalizations of migrant patients compared to natives in a well-defined area of the metropolitan city of Bologna and evaluate the effect of the Covid 19 pandemic on the incidence of psychiatric hospitalizations among migrants and on their clinical characteristics.

**Methods:**

The study conducted is of an observational and retrospective type on migrant and native patients admitted to the psychiatric unit “SPDC-Malpighi” of the DSM-DP of Bologna AUSL between 01/01/2018 and 31/12/2020.

**Results:**

Migrants were more likely to be admitted via ED and less likely to be referred from a CMHC or from non-psychiatric hospital unit compared with natives. Most migrants were discharged at home while natives more frequently chose to self-discharge. With regard to diagnosis, migrants were more likely to be admitted due to a SSD, while natives were more likely to be diagnosed with a MD or SUD.

**Conclusions:**

We confirm the presence of differences in access to care, type of discharge and type of diagnosis between migrants and natives. Further studies to investigate changes in pre and post Covid admissions in migrants would be needed.

**Disclosure:**

No significant relationships.

